# Synthesis of Graphene Oxide Interspersed in Hexagonal WO_3_ Nanorods for High-Efficiency Visible-Light Driven Photocatalysis and NH_3_ Gas Sensing

**DOI:** 10.3389/fchem.2019.00722

**Published:** 2019-11-01

**Authors:** Tarek M. Salama, Mohamed Morsy, Rabab M. Abou Shahba, Shimaa H. Mohamed, Mohamed Mokhtar Mohamed

**Affiliations:** ^1^Department of Chemistry, Faculty of Science, Al-Azhar University, Cairo, Egypt; ^2^Building Physics and Environment Institute, Housing and Building National Research Center (HBRC), Giza, Egypt; ^3^Department of Chemistry, Faculty of Science (Girls Branch), Al-Azhar University, Cairo, Egypt; ^4^Chemistry Department, Faculty of Science, Benha University, Benha, Egypt

**Keywords:** hexagonal WO_3_, graphene oxide, hydrothermal method, gas sensing, MB photocatalysis, visible light irradiation

## Abstract

WO_3_ nanorods and GO (at 1 wt% loading) doped WO_3_ were synthesized using a template free deposition-hydrothermal route and thoroughly characterized by various techniques including XRD, FTIR, Raman, TEM-SAED, PL, UV-Vis, XPS, and N_2_ adsorption. The nano-materials performance was investigated toward photocatalytic degradation of methylene blue dye (20 ppm) under visible light illumination (160 W, λ> 420) and gas sensing ability for ammonia gas (10–100 ppm) at 200°C. HRTEM investigation of the 1%GO.WO_3_ composite revealed WO_3_ nanorods of a major *d*-spacing value of 0.16 nm indexed to the crystal plane (221). That relevant plane was absent in pure WO_3_ establishing the intercalation with GO. The MB degradation activity was considerably enhanced over the 1%GO.WO_3_ catalyst with a rate constant of 0.0154 min^−1^ exceeding that of WO_3_ by 15 times. The reaction mechanism was justified dependent on electrons, holes and •OH reactive species as determined via scavenger examination tests and characterization techniques. The drop in both band gap (2.49 eV) and PL intensity was the main reason responsible for enhancing the photo-degradation activity of the 1%GO.WO_3_ catalyst. The later catalyst initiated the two electron O_2_ reduction forming H_2_O_2_, that contributed in the photoactivity improvement via forming •OH moieties. The hexagonal structure of 1%GO.WO_3_ showed a better gas sensing performance for ammonia gas at 100 ppm (R_a_-R_g_/R_g_ = 17.6) exceeding that of pure WO_3_ nanorods (1.27). The superiority of the gas-sensing property of the 1%GO.WO_3_ catalyst was mainly ascribed to the high dispersity of GO onto WO_3_ surfaces by which different carbon species served as mediators to hinder the recombination rate of photo-generated electron-hole pairs and therefore facilitated the electron transition. The dominancy of the lattice plane (221) in 1%GO.WO_3_ formed between GO and WO_3_ improved the electron transport in the gas-sensing process.

## Introduction

The nanoscience and nanotechnology have allowed the development of nanosized materials of unique electronic and optical properties quite different from those of their bulk states (Pang et al., 2010). Metal nanoparticles were recognized to reveal sum of unique optical and electronic properties, which result largely from surface plasmon resonance phenomenon. However, the high cost and low stability following annealing usually limits their applications. Nevertheless, composite materials including metal oxide nanoparticles have drawn great attention due to their unique chemical and physical properties, which make them applicable for use in photocatalysis and gas sensing (Bittencourt et al., 2006; Guo et al., 2012; Chen et al., 2013). Among the existing metal oxides, tungsten oxide (WO_3_) as an n-type semiconductor of a band gap of 2.5–2.8 eV owns important applications in numerous fields (Zeng et al., 2012; Gui et al., 2015; Behera and Chandra, 2018). WO_3_-based nanomaterials with various morphologies have been widely investigated for chemical gas sensors (Chu et al., 2017; Kaur et al., 2018; Gao et al., 2019) such as for detecting NH_3_, H_2_, and ethanol (Tsai et al., 2017; Chen et al., 2018; Morsy et al., 2018, 2019). Besides, it has attracted a lot of interest in photocatalysis theme because of its strong adsorption, manipulated energy band gap and visible light absorption (Zhang et al., 2017). However, the application of WO_3_ as gas sensors is reduced by the bad selectivity, long response time, low sensitivity and high resistivity (Urasinska-Wojcik et al., 2016). Generally, nanocrystalline semiconductors have poor charge mobility and thus produce very limited photocurrent. This regards as one of the biggest obstacle that hinder the usage of WO_3_ as practical photocatalysts. Thus, many attempts have been made to improve the behavior of WO_3_ such as morphology control, surface hybridization and forming hybrid composites (Li et al., 2015; Galstyan et al., 2016; Shendage et al., 2017). Improved photocatalytic activities and gas sensing performances have been demonstrated for WO_3_-graphene composite. Recently, graphene-based composites have received considerable attention due to their potential applications in many useful fields including photocatalysis (Zhang et al., 2015; Luna et al., 2018) and gas sensors (Behera and Chandra, 2018). The enhanced performance was based on the composite high conductivity, large surface area and the P-type conductivity created due to adsorbed oxygen molecules localized on graphene structure. With attaching an n-type semiconductor, it might be feasible to boost the reaction activity based on the facile charge transfer via the p-n junction interface. Expectedly, graphene not only can facilitate the nucleation and growth of the nanocrystals but also help achieving its stability and size beside it can orientate the oxide crystal growth (Zhang et al., 2015; Quan et al., 2017).

For the purpose of optimizing WO_3_ nanoparticles for improving ammonia gas sensing and MB photocatalytic degradation, the surface hybridization of WO_3_ with a small loading of GO was carried out. Thus, GO at 1 wt% loading was successfully dispersed in the matrix of WO_3_ hexagonal structure as synthesized by the *in-situ* deposition hydrothermal technique. The 1%GO.WO_3_ and WO_3_ free GO catalysts were well-characterized using various techniques including XRD, TEM-SAED, UV-Vis, PL, FTIR, Raman, XPS, and N_2_ sorptiometry. The catalysts were then tested as visible light photocatalysts toward methylene blue (MB) degradation and as gas sensors for NH_3_ gas.

## Experimental

### Materials and Methods

Sodium tungstate dihydrate (Nice Chemicals, India), hydrochloric acid (Fisher Scientific, UK), oxalic acid (Adwic, Egypt), potassium permanganate, and sulfuric acid (Sigma-Aldrich, Germany), sodium nitrite (BDH Prolabo chemicals, USA), graphite (Merck, Germany), hydrogen peroxide (GFS Chemicals, USA), and methylene blue dye (S.D. Fine-Chem Limited, India) were used as obtained.

### Synthesis of WO_3_ Nanorods

Sodium tungstate (6.6 g, 0.2 mol) was dissolved in 100 ml distilled water and then acidified with HCl till pH 1, to form an immediate white precipitate. This precipitate was dissolved in 30 ml distilled water containing 0.4 g oxalic acid to obtain clear and transparent solution. The final solution was transferred to 40 ml Teflon stainless steel autoclave and maintained at 180°C for 26 h. After reaction, the product was washed several times with distilled water and ethanol to remove any unreacted residuals and finally dried at 100°C for 1 h.

### Synthesis of Graphene Oxide-Loaded Tungsten Oxide Nanorods

A typical synthesis procedure of GO loaded onto WO_3_ nanorods with a GO loading of 1% by weight was as follows. A definite amount of GO was dissolved in 50 ml distilled water to form a pasty brownish color solution. To this solution, a proper sodium tungstate (6.6 g, 0.2 mol) solution was slowly added and the pH of the mixture was adjusted to 1 by addition of aqueous HCl. Then, 0.4 g of oxalic acid dissolved in 30 ml water was added and stirred continuously at room temperature for 3 h. The latter mixture was transferred into a 250 ml Teflon stainless steel autoclave and maintained at 180°C for 26 h to get the final product. This catalyst is denoted as 1%GO.WO_3_.

### Physical Methods and Analysis

The powder X-ray diffraction (XRD) patterns were recorded, on Ni-filtered copper radiation (λ = 1.5404 Å) at 45 kV and 40 mA with a scanning speed of 2θ = 2.5°/min, on a Bruker diffractometer, type D8 ADVANCE (Germany). The TEM micrographs were measured with a FEI Tecnai G20 Super-Twin microscope (USA), at an accelerating voltage of 200 kV. The powder samples were put on carbon foils with a microgrid and the TEM images were measured with minimum electron irradiation to prevent the samples damage, together with the selected area electron diffraction patterns. Fourier transform infrared (FT-IR) spectra were recorded via a single beam Perkin-Elmer Spectrometer (RXI, USA) with a resolution of 2 cm^−1^ in the region 4,000–400 cm^−1^. Raman spectra were obtained with a Bruker Senterra Raman spectrometer (Germany), using the 532 nm line as the excitation beam. The incident laser power and resolution were 5 mW and 2 cm^−1^, respectively. The surface properties namely BET surface area, total pore volume (V_p_) and mean pore radius (r^−^) were determined from the nitrogen adsorption isotherms measured at −196°C using a Nova 3200 porosimeter, USA. The solid samples were out-gassed at 473 K for 2 h under a reduced pressure of 10^−4^ Torr before starting the measurements. Diffuse Reflectance UV–Vis spectra (UV–Vis DRS) were recorded using a UV–Vis JASCO spectrophotometer (V-570, Japan) in the range of 200–1,000 nm. The edge energy (E_g_) was determined by finding the intercept of the straight line in the low-energy rise of the plot of [F(R_∞_)hν]^2^, for the direct allowed transition vs. hν, where hν is the incident photon energy. The photoluminescence (PL) excitation and emission spectra were measured on a FL/FS 900 time resolved fluorescence spectrometer. The measurements were conducted at room temperature using a He-Cd laser (310 nm), as an excitation source. X-ray photoelectron spectroscopy (XPS) equipped with an Al-Kα x-ray source at energy of 1486.6 eV was utilized to distinguish the surface chemical composition of the catalysts. Electrochemical measurements were determined using Digi-Ivy 2116 B, USA, in 6.0 M KOH aqueous solution for determining either N_D_ or N_A_ concentration using the Mott-schottky plot. More details information about the measurement conditions can be found elsewhere (Mohamed et al., 2017).

### Photocatalytic Degradation Experiments

The photocatalyst (100 mg) was suspended in 100 mL aqueous solution containing 20 mg L^−1^ methylene blue (MB). For each experiment, the suspension was stirred in dark for 60 min to establish the adsorption–desorption equilibrium between the MB and the catalyst, followed by irradiation with a high-pressure Philips lamp of 160 W with an average light intensity of 60 mWcm^−2^, emitting only visible light (400–700 nm) via using a cut-off filter. Three ml aliquot containing dye and catalyst powder was taken out followed by centrifuging at 3,000 rpm for 5 min. After the catalyst separation, the change in MB concentration was determined through the absorbance variation at the wavelength of 664 nm. The absorption spectra of the aqueous solution of the MB after adsorption and degradation were measured using a Shimadzu UV-2350 spectrophotometer, Japan. The reactive species were also determined to stand on the degradation mechanism via employing various scavengers.

### Sensor Fabrication and Evaluation

Fluorinated tin oxide (FTO) glass substrate has been used for sensors fabrication. In a typical procedure, 1 mm gap was created on the conductive FTO substrate using an electric arc followed by washing/cleaning processes via soaking in a soap solution for 10 min, flowed by distilled water and acetone. Finally, the FTO substrate was dried under N_2_ flux. The electrical contacts were established via attaching two copper foils with conductive adhesive onto the FTO substrate. A proper amount of the GO.WO_3_ composite was mixed with deionized water in a mortar forming a paste. The paste was then applied onto the FTO substrate and annealed at 80°C for 4 h. The sensor was aged at 5 V for 36 h to enhance its stability. Following stabilization of the sensor resistance, a known concentration of ammonia gas was generated by injection into a fixed volume chamber supplied with heater. The ammonia gas concentration in ppm was calculated using the following equation: ***Vi*** **=** ***C.V.MW/*22.45** ***D***, Where *MW* is the molecular weight of ammonia, *D* is the density of ammonia, *V* is the chamber volume, *C* is the concentration of ammonia in ppm, and *Vi* is the injected volume of ammonia. The real time resistance variation was recorded every 3 s by a digital precision pico-ammeter type keithley-6487 interfaced to a computer. The response of the sensor (R_a_- R_g_/R_g_) ^*^ 100 was defined as the ratio of the resistances of the sensor in air (R_a_) to that in ammonia (R_g_). The response time was defined as the time taken by the sensor to achieve 90% of the total resistance changes.

## Results and Discussion

### X-Ray Diffractometry (XRD)

**Figure 1:**
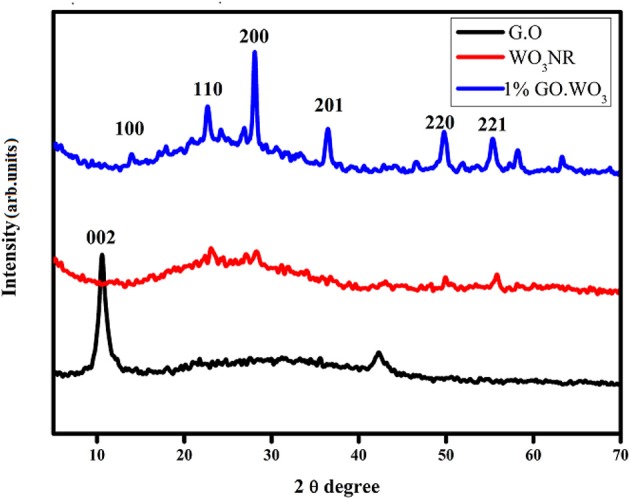
XRD patterns of GO, WO_3_, 1%GO.WO_3_ catalysts.

Figure 1 illustrates the XRD patterns of the WO_3_ nanorods, graphene oxide (GO) and WO_3_ nanorods doped with 1% GO. As for GO, an intense reflection related to GO appears at 2θ = 10.64° diffracted from the (002) plane. By applying Bragg's law, the calculated interlayer spacing of GO is 0.83 nm, providing an expansion than graphite analog depicted at 0.34 nm. This is interpreted in terms of elongation of the c-axis during graphite oxidation to GO via creation of oxygen-containing functional groups. The pure WO_3_ sample is well-crystallized in a single phase with exposing diffraction peaks at 2θ equal 24.68°, 28.17°, 36.52°, 49.85°, and 55.43°, indexed to hexagonal WO_3_ (JCPDS 85-2459). The average crystallite size of WO_3_ determined via Scherer's equation is 20 nm. All reflections due to the 1%GO.WO_3_ pattern are associated with a single phase hexagonal WO_3_. This sample exhibits more intense reflections when compared with the GO free WO_3_ sample, endorsing an average crystallite size of 25 nm. Besides, the peak of GO disappears in the 1%GO.WO_3_ pattern, which is likely due to the thimbleful of GO in the composite. However, the revealed broadness in the latter pattern for the peak at 24.68°, along with the expansion in d values is indicative of the dispersion of GO into the WO_3_ hexagonal structure.

### High-Resolution Transmission Electron Microscopy (HRTEM)

**Figure 2:**
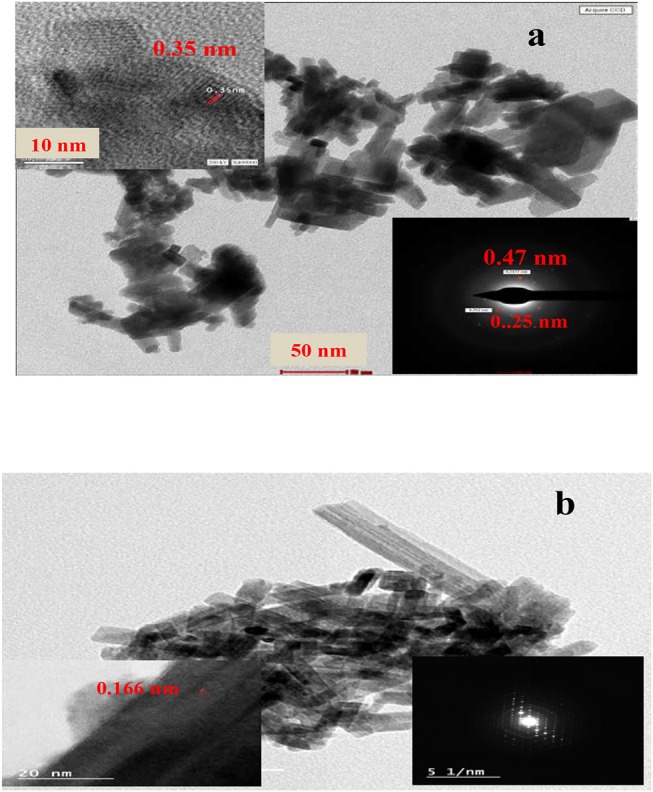
TEM, HREM, and SAED (inset) images of GO inset in **(a)** WO3 **(a)** 1%GO.WO3 **(b)** catalysts.

Particle morphology, crystallinity and selected area electron diffraction (SAED) analyses of the synthesized samples are studied by means of HRTEM (Figure 2). The HRTEM image of WO_3_ shows that it owns nanorods shape with an average diameter of 4.11 nm. The magnified HRTEM image in the inset of the WO_3_ Figure shows good crystallinity and spacing's of well-resolved lattice fringes at ~0.35 nm, coincidently matching the (110) diffraction plane of WO_3_ (JCPDS 85-2459). On the other hand, the SAED pattern of this sample shows a distorted hexagon with d = 0.24 nm corresponding to the (201) plane of WO_3_ nanorods. The HRTEM image of the 1%GO.WO_3_ composite shows clear nanorods with an average diameter of 17.6 nm and lattice spacing's equal 0.16 nm due to the (221) plane. This plane is not apparent in pure WO_3_ nanorods. The SAED pattern in the inset of the latter Figure shows six-fold symmetry diffraction bright spots appears as well-defined diffraction hexagonal form, unlike that of pure WO_3_ nanorods, emphasizing the interaction of GO with WO_3_.

### FTIR Spectra

**Figure 3:**
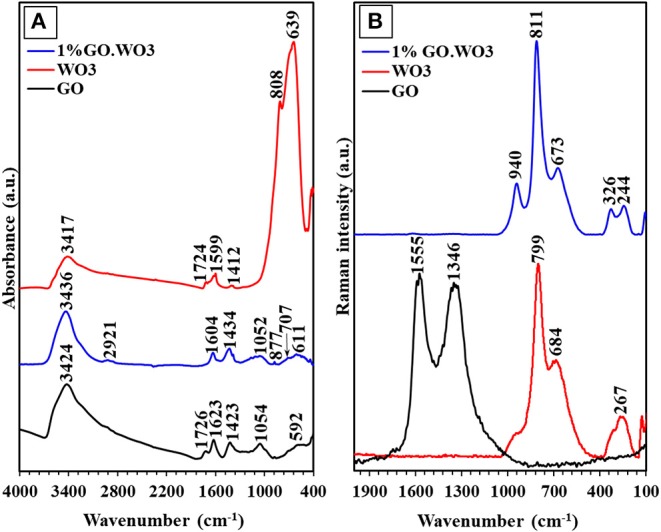
FTIR spectra **(A)** and Raman spectra **(B)** of GO, WO_3_, 1%GO.WO_3_ catalysts.

The interaction between WO_3_ and GO is confirmed by the FTIR spectroscopy as exemplified in Figure 3A. The FTIR spectrum of GO exhibits a broad band with a maximum at 3,424 cm^−1^ corresponding to the OH stretching vibration (ν_OH_). The substantial OH band broadening is a result of the strong hydrogen bonding interaction of hydroxyl containing groups, while the small band at 1,719 cm^−1^ is due to the C=O stretching vibration from the residual COOH groups on the GO surfaces. The sp^2^ carbon network revealed from the band at 1,623 cm^−1^ is assigned to the C=C skeletal asymmetric vibration of unoxidized graphitic domains (Poongodi et al., 2017). The bands at 1,423 and 1,054 cm^−1^ correspond to the stretching vibrations of the C-OH and C-O groups, respectively (Shendage et al., 2017). For WO_3_ nanorods, the small broad band observed at 3,417 cm^−1^ corresponds to the OH stretching, and the strong bands at 808 and 639 cm^−1^ to the stretching vibration of O-W_b_-O and O-W_c_-O links in the WO_3_ structure, respectively (Han et al., 2018). The peak related to the W=O stretching vibration, proposed for dangling oxygen bonds, is obscured by the strong absorption of the former peak. The 1%GO.WO_3_ composite shows a band at 3,436 cm^−1^ ascribed to the OH stretching vibration. By comparing with that of GO, this band becomes stronger, narrower and shifts to a higher frequency in the spectrum of 1%GO.WO_3_ composite, i.e., from 3,424 to 3,436 cm^−1^. This reflects the interspersion of GO matrix within the WO_3_ structure (Zhang et al., 2010). In addition, the carbonyl C=O band at 1,719 cm^−1^ is disappeared in the spectrum of 1%GO.WO_3_. This result implies that the carboxylic functional groups of GO are involved in the GO.WO_3_ interactions (Saidi et al., 2018). The peaks due to O–W–O stretching vibrations are decreased in intensity and red shifted to 707 and 611 cm^−1^, inferring the GO dispersion into the WO_3_ hexagonal structure (Saidi et al., 2018). A small band at 877 cm^−1^ characteristics of the stretching W=O vibration is noticed, which never seen in the GO free WO_3_ (Saidi et al., 2018). This indeed confirms the strong interaction between the two-component forming composite in which GO contributes in the formation of W=O bonds. The GO doping is rather confirmed from the presence of peaks assignable to C=C and C-OH stretching vibrations at 1,604 and 1,434 cm^−1^, respectively. In this case, it is observed that the shift of bands at 1,604 and 1,434 cm^−1^ in 1%GO.WO_3_ toward higher and lower values when compared with those for individual GO (1,623 and 1,423 cm^−1^), respectively, reflects the strong interaction between the components forming the composite. In addition, there is almost no change in the peak at 1,052 cm^−1^ both in GO and 1%GO.WO_3_ confirming that C-O groups did not take part in the reaction between GO and WO_3_, unlike COOH and C-OH functional groups.

### Raman Spectra

Figure 3B shows the Raman spectra of GO, WO_3_ nanorods and 1%GO.WO_3_. The GO spectrum exhibits two intense bands at 1,346 and 1,559 cm^−1^; the former D-band represents the disordering sp^2^-hybridized C atoms of GO, while the latter G-band corresponds to the structural integrity of the sp^2^-hybridized C atoms of GO (Zeng et al., 2012). The Raman spectrum of pure WO_3_ nanorods is characterized by two bands at 799 and 684 cm^−1^ assigned to the W-O-W stretching vibrations, which according to previous reports signified to the distances of the W-O bonds in pure WO_3_ (Gao et al., 2019). The shoulder near 920 cm^−1^ is assigned to the stretching frequency of the terminal W=O on the nanostructural boundaries of WO_3_ (Kaur et al., 2018), whereas Raman frequencies at 326 and 267 cm^−1^ are most likely due to the O-W-O deformation modes. The Raman spectrum of 1%GO.WO_3_ is only dominated by the W-O-W stretching modes at 799 and 684 cm^−1^ (Figure 3B), and their relevant deformation ones occurred at 326 and 244 cm^−1^. It is observed that the molecular structure of GO (D- and G-bands) is vanished after inclusion of GO onto the WO_3_ nanorods. This agrees with the low content of GO (1 %) in the sample. The Raman peaks of 1%GO.WO_3_ at 940 and 811 cm^−1^ are weakened and rather shifted to higher frequencies as compared to 920 and 799 cm^−1^ for WO_3_, advocating strong interaction between WO_3_ and GO matrix. Similarly, developing of two peaks at 244 and 326 cm^−1^ in 1%GO.WO_3_ contrary to only one peak at 267 cm^−1^ in WO_3_, features a distortion in the nanostructured WO_3_ (shortening O-W-O bonds) revealing its strong bonding with GO, as typically confirmed from the XRD analysis of 1%GO.WO_3_.

### N_2_ Sorptiometry

**Figure 4:**
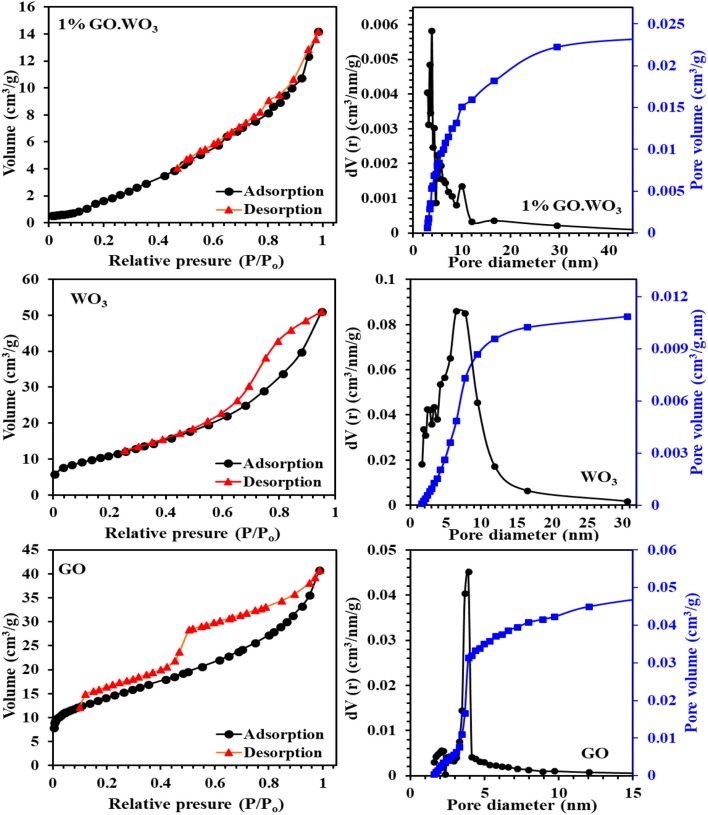
Adsorption-desorption isotherms and pore size distribution curves (in-set) of GO, WO_3_, 1%GO.WO_3_ catalysts.

Figure 4 shows the adsorption-desorption isotherms and pore size distribution curves (inset) of all the samples. The WO_3_ nanorods isotherm fits type IV of mesoporous materials, in agreement with the IUPAC assembling. The adsorption-desorption coincides at P/P_o_ of 0.5, revealing the existence of wide pores with H4 type. Where in 1%GO.WO_3_ the adsorption-desorption isotherm coincides at P/P_o_ of 0.46 indicating narrowing of the pores as a result of GO incorporation within the WO_3_ pores. In concordance, the pore radius of WO_3_ nanorods indicates a value of 6.5 nm where it becomes narrower for 1%GO.WO_3_ at 3.9 nm. The pore size distribution (PSD) of WO_3_ nanorods in the inset of Figure 4 manifests a monomodal type of pore maximized at 7.5 Å that extends to 20 Å. Contrarily, the 1%GO.WO_3_ indicates PSD at 4.0 Å, proposing the evolution of more narrow pores, to comprehend the role of GO in narrowing the pore mouth of the WO_3_ hexagonal array. The surface area of WO_3_ nanorods (54.3 m^2^/g) exceeds that of the 1%GO.WO_3_ composite (14.2 m^2^/g); a concurrent trend involving a lager pore volume for the former (0.084 cm^3^/g) than for the latter (0.024 cm^3^/g). This result emphasizes the inclusion of GO precursor into the WO_3_ nanorods.

### Optical Characteristics

**Figure 5:**
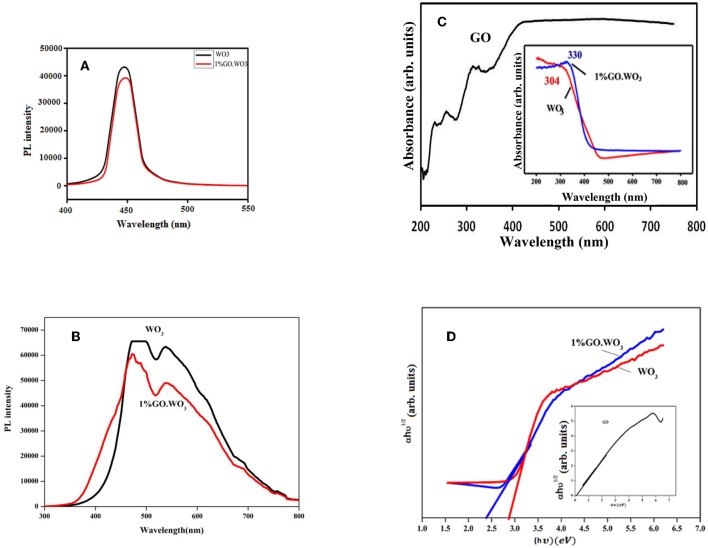
Photoluminescence (PL) emission spectra of WO_3_ and 1%GO.WO_3_ in the margin of 400–550 nm **(A)**, WO_3_ and 1%GO.WO_3_ in the margin of 300–800 nm all excited at 320 nm **(B)**, beside UV-visible spectra **(C)**, and gap energy plots **(D)** of the GO (inset), WO_3_, 1%GO.WO_3_ catalysts.

Figures 5A,B present the PL emission spectra of WO_3_ NRs and 1%GO.WO_3_ excited at 320 nm at different wavelengths margin. A clear main broad emission peak at 447 nm is observed in the PL spectra of both samples. Based on previous investigations in literature (Jung et al., 2010; Chu et al., 2017), this peak may originate from the presence of oxygen vacancies or defects in WO_3_ NRs. The decrease in PL emission intensity at 447 nm for 1%GO.WO_3_ compared to that for WO_3_ NPs (Figure 5B) is likely a consequence of an electron transfer from the WO_3_ conduction band to GO networks. Such quenching of PL emission of 1%GO.WO_3_ discloses that GO could acts as an electron transfer channel in the GO-encompassed semiconductor materials, as indicated in previous studies (Min et al., 2012; Dong et al., 2014). GO can thus virtually lower the rate of the electron-hole recombination, leading to maximizing charge separations and conserving more reactive species for high photocatalytic activity. Another emission peak at 560 nm is seen in Figure 5B for both samples however, the peak of 1%GO.WO_3_ was of lower intensity reflecting the decrease in the electron-hole recombination.

Figure 5C shows the UV–Vis absorption spectra of WO_3_ NRs and 1%GO.WO_3_ (inset) in comparison with GO spectrum. The latter spectrum indicates bands at 250, 280 and 320 nm comprehending the presence of π-π^*^ and n-π^*^(C=O), respectively. This indeed indicates the presence of both graphene and graphene oxide structures. The WO_3_ NRs, and 1%GO.WO_3_ samples exhibit wide absorption bands at 304 and 330 nm, respectively. Meanwhile, the latter reveals an absorption edge at 430 nm, which is lower in energy than the relevant of 1%GO.WO_3_ at 480 nm. This could give an indication of decreasing the oxygen deficiencies on WO_3_ NRs as consequence of GO incorporation. Besides, the 1%GO.WO_3_ catalyst displays absorption in the visible region from 400 to 700 nm stronger than that of free WO_3_, signifying the presence of W-O-C linkages in the composite. This manifests the expected photocatalytic capability of 1%GO.WO_3_ under visible light irradiation. The band gaps of the catalysts calculated from the absorption spectra using the Tauc draw (Figure 5D) are 2.49, 2.8, and 0.55 eV for 1%GO.WO_3_, WO_3_ NRs, and GO, respectively. Indeed, the incorporation of GO might led to the decrease in the bond length of W=O and/or O-W-O, possibly due to the strong interaction between WO_3_ and GO and via elaborating bonds such as C-O-W and C-W.

### XPS Study

**Figure 6:**
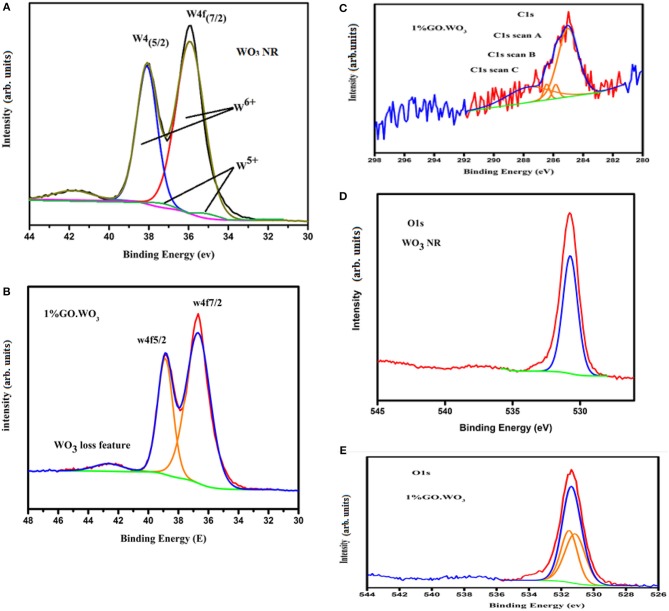
W 4f XPS spectra of WO_3_ **(A)** and 1%GO.WO_3_ **(B)**, beside the C1S of 1%GO.WO_3_ **(C)**, and O1S of WO_3_ **(D)** and 1% GO.WO_3_ **(E)** catalysts.

XPS, illustrated in Figure 6, is used to have an idea about WO_3_ chemical composition as well as tracing the changes in WO_3_ chemical states following GO incorporation. XPS survey scan (not shown) verifies the presence of W, C and O elements in both pure WO_3_ nanorods and 1%GO.WO_3_ samples. The core-level W4f XPS of WO_3_ nanorods (Figure 6A) shows the presence of strong two peaks at 35.92 and 38.09 eV together with their satellite band at 41.88 eV due to the spin-orbit split levels of W(4f7/2) and W(4f5/2), respectively. The spectral deconvolution indicates two subpeaks at 35.4 and 37.3 eV due to W^5+^ state. The high-resolution W4f spectrum of 1%GO.WO_3_ (Figure 6B) shows two well-defined feature peaks located at 36.6 and 38.8 eV to fit W4f7/2 and W4f5/2, consistent with W^6+^ state in the form of WO_3_ together with a loss feature at 42.6 eV of W(4f3/2) (Naseri et al., 2011). The latter confirms the successful incorporation of GO into WO_3_ structure, manifested by the exhibited shift of all W4f peaks into higher binding energies. Also, this shift indicates that the low valence W^5+^ state seen in WO_3_ nanorods is no longer detectable in 1%GO.WO_3_ featuring the absence of substoichiometric WO_3−x_ following the GO incorporation.

The high-resolution C 1 s spectrum (Figure 6C) of 1%GO.WO_3_ owns a strong peak of C=C species positioned at 285.2 eV. This spectrum is also divided into three bands at 285.84, 286.45, and 287.69 eV attributed to C-C, C-O, and C=O groups, respectively. The surface atomic percentages show that C=C represents the highest ratio (62.2%) reflecting a reduction in the oxygen content of GO in 1%GO.WO_3_. The latter deduction is justified by ratios of C=O (29.46%), C-C (3.93%) and C-O (4.39%). This result verifies the successful formation of a hybridized structure of GO.WO_3_.

The normalized XPS O 1 s spectrum shown in Figure 6D of WO_3_ NRs shows one peak at BE of 530.73 eV, assigned to the ${\text{O}}_{2}^{-}$1s ion (lattice oxygen). The XPS spectrum of the O 1 s of 1%GO.WO_3_ (Figure 6E) is splitted into two component peaks localized at 531.51 and 531. 15 eV ascribed, respectively, to O1s O-C and ${\text{O}}_{2}^{-}$ states, which represent a total surface oxygen moiety of 53.89 vs. 100% at the surfaces of WO_3_ NRs. This indeed advocates the GO involvement in the WO_3_ structure. Conclusively, the majority of GO is reduced to graphene (62.2%) with exposing residual oxides correlated to C=O (29.46%) and C-O (4.39%) moieties on GO amounted to the promotion of P-type conducting nature. This is capable of forming conducting transition when amalgamated with WO_3_ (n-type) to boost the charge transfer via the p-n junction interface and thus the reaction activity.

### Photocatalytic Degradation of MB

**Figure 7:**
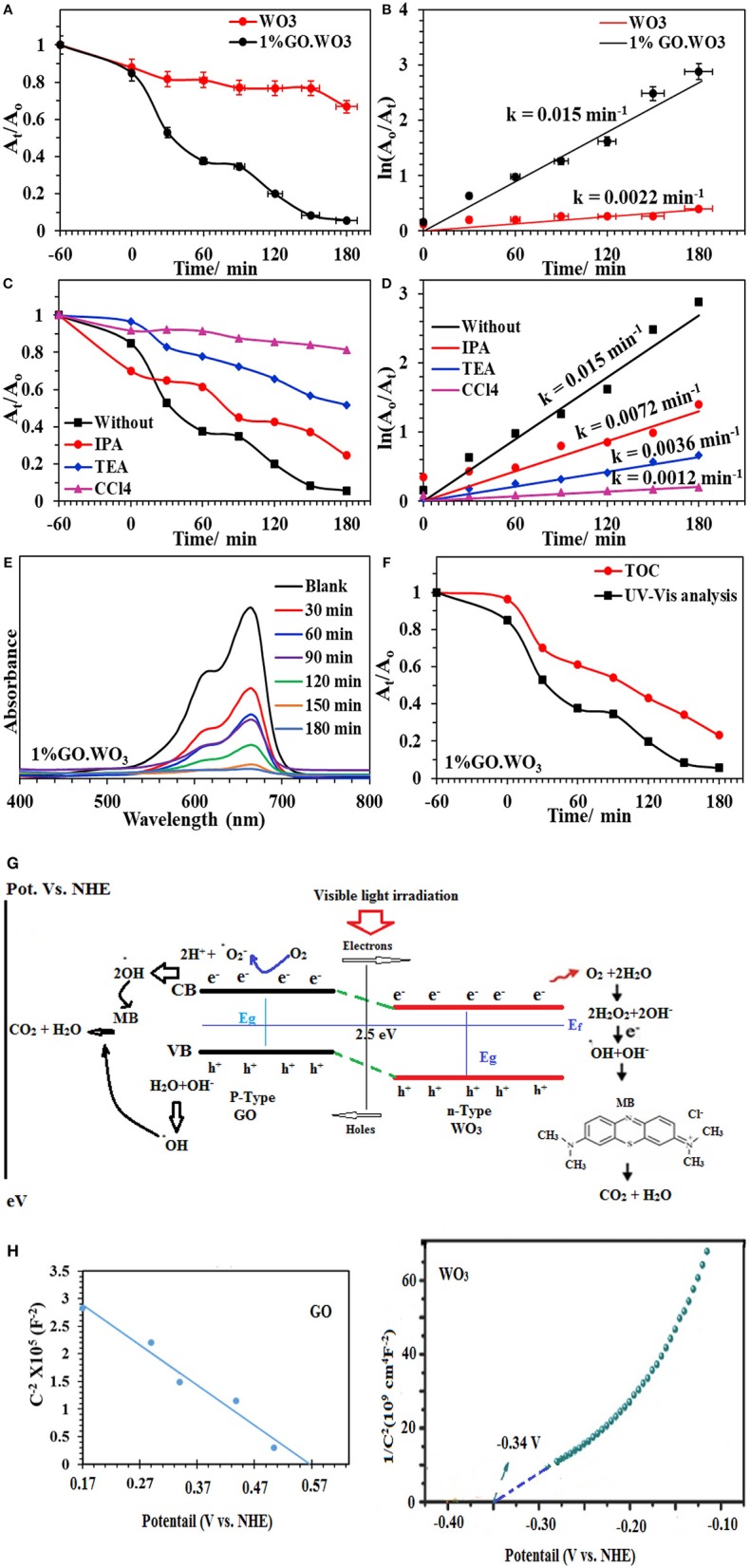
A_t_/A_o_ plot of the MB (20 ppm) degradation over WO_3_ and 1%GO.WO_3_ catalysts at the dose of 1.0 g/l under visible light illumination (160 W, λ > 420) **(A)** and their kinetic curves of ln A_o_/A_t_ vs. time **(B)** Effect of scavengers on the catalytic degradation of MB over 1%GO.WO_3_ with time **(C)** and their kinetic curves with time **(D)** beside UV-Vis. absorbance spectra of the MB over 1wt% GO.WO_3_ with time **(E)**, Decrease in TOC and change in absorbance as function of irradiation time for the MB dye **(F)**, the expected reaction mechanism of the MB degradation over 1%GO.WO_3_ **(G)** and Mott–Schottky plots of WO_3_ and GO **(H)**.

The photocatalytic activities of WO_3_ NRs and 1%GO.WO_3_ toward the degradation of methylene blue dye (MB; 20 ppm) under visible light illumination are shown in Figure 7A. Prior irradiation, the suspension formed between the catalyst and the dye is stirred in dark for 1 h to ensure diffusion from the bulk of dye to the interfacial zone and to accomplish adsorption-desorption equilibrium between them. The MB adsorption ability of 1%GO.WO_3_ increases over 17% above WO_3_ NRs, elaborating that GO-doping of WO_3_ with small amount as 1% does not effectively improve the electrostatic attraction between MB and the catalyst. The result in Figure 7A shows an enhanced degradation of the MB dye over 1%WO_3_.GO under visible light irradiation through a complete degradation (100%) in 180 min at a rate constant of 0.0154 min^−1^; as shown in Figure 7B, in front of 34% degradation with a rate of 0.001 min^−1^ for WO_3_ NRs. Figure 7C shows the UV-vis spectra of the MB photodegradation over 1%GO.WO_3_ as a function of elapsed time elucidating the consequences of the MB degradation. These results indicate that the marked increase in activity of the 1%GO.WO_3_ catalyst is explained by the slight increase in the visible light absorption beside lowering both the band gap and the PL intensity; reflecting the slow recombination between electrons and holes. Also, the high dispersity of GO in WO_3_ NRs; as emphasized by the FTIR, Raman and XPS data, evokes the involvement of carbons via hindering the recombination rate of photo-generated electron-hole pairs within the WO_3_ hexagonal structure, facilitating the electron transition. Performing the MB degradation over WO_3_ incorporated higher GO weight percentages (such as 5 and 10%) indicates adsorption behavior rather than photocatalysis trend (not shown). That is why we were only restricted to the 1%GO ratio. Executing the MB degradation (20 ppm) over 1%GO.WO_3_ in presence of various scavengers under visible light illumination (Figure 7D) results in a significant decrease in the rate constant, compared to 0.0124 min^−1^; evaluated in the absence of any scavenger. From Figure 7D, the maximum decease in the rate constant is for that depicted for the CCl_4_ scavenger (0.0008 min^−1^), implying that electrons are the most reactive species affecting the MB degradation, followed by triethanolamine (0.0029 min^−1^) and isopropanol (0.0053 min^−1^), configuring in sequence the importance of holes and •OH after electrons. Figure 7E depicts the UV-Vis spectra of the MB dye as a function of time in the presence of the 1%GO.WO_3_ photocatalyst. A magnificent decrease after 180 min is well-illustrated. Interestingly, the enhanced photocatalytic activity for photodegradation of MB is acquired over WO_3_ modified low conc. of GO (1%) of eco-friendly synthesis conditions. To identify the extent of MB mineralization, Figure 7F shows the reduction in TOC in comparison to the dye degradation curve. Apparently, the TOC curve decreases significantly to 77% in 180 min confirming the presence of none degradable products and rather affirming the high mineralization capability of the catalyst.

The above mentioned results suggest that electrons, holes and •OH are the main reactive species in the process of MB photodegradation over the 1%GO.WO_3_ catalyst. This is not mean the insignificant effect of holes but it works as an indirect way as elaborated in the mechanism to deliver ^•^OH. Based on the above outcomes, a suggested mechanism for the photocatalytic reaction is depicted as in the scheme shown in Figure 7G. Under visible light irradiation, the valence band (VB) electrons are excited to the conduction band (CB) of the WO_3_ semiconductor, creating holes (h^+^) in the VB. The conduction band (CB) edge and valence band (VB) edge can be calculated by the following equations (Sadakane et al., 2010):

(1) $$\begin{array}{l} EVB=X-E^{\text{e}}+1/2Eg \end{array}$$

(2) $$\begin{array}{l} ECB=EVB-Eg \end{array}$$

Where EVB and ECB are the valence band and conduction band edge potentials respectively where χ is the geometric mean of electronegativity of the constituent atoms semiconductor. The values of χ for GO.WO_3_ and WO_3_ are calculated as 6.39 eV and 6.59 eV according to the literature (Pearson, 1988). *E*^e^ is the energy of free electrons vs. hydrogen (4.5 eV) scale and *E*g is the band gap energy of the semiconductor. A Mott-Schottky analysis is constructed to examine the carrier density and flat band potential for both WO_3_ and GO. The *E*_fb_ is estimated by extrapolating each Mott–Schottky plot to the *x*-axis to obtain the intercept value and the *N*_d_ is estimated by the slope following Equations 3 and 4 (Mohamed et al., 2018a,b);

(3) $$\begin{array}{l} 1/C^{2}=\left[2/\left({e\varepsilon \varepsilon_{0}N}_{\text{d}}\right)\right]\left(E-E_{\text{fb}}-\kappa T/e\right) \end{array}$$

(4) $$\begin{array}{l} N_{\text{d}}=2/\left(e\varepsilon \varepsilon_{0}\right){\left[d\left(1/C^{2}\right)/d\left(E\right)\right]}^{-1} \end{array}$$

Where *C* is the capacitance of the space charge layer, *N*_d_ is the number of donors, *e* (1.602 × 10^−19^ C) is the electron charge, ε (20 for WO_3_) is the dielectric constant, and ε_0_ (8.85 × 10^−14^ F cm^−1^) is the vacuum permittivity, and κ (1.38 × 10^−23^ J K^−1^) is the Boltzmann constant. First, the positive slopes indicate the n-type nature of the WO_3_ samples (Figure 7H). Moreover, the calculated *N*_d_ is 1.30 × 10^20^ cm^−3^, thus enormously promoting the charge transport efficiency. In the meantime, the flat band potential equal −0.34 V. The flat band potential of GO is measured at 0.57 V. Assuming that majority of the depletion width is located in GO film, the depletion width in the 1%GO.WO_3_ interface is estimated according to following equation:

(5) $$\begin{array}{l} V_{bi}\left(\text{p}\right)=qN_{\text{A}}{\text{x}}_{\text{p}}^{2}/2\varepsilon \text{GO} \end{array}$$

Where *N*_A_, ε and εGO are acceptor concentration and absolute permittivities of WO_3_ and GO respectively. Of particular interest, GO shows a negative slope corresponding to p-type conductivity manifesting the presence of high oxygen vacant defects with carrier concentration (N_A_-holes) of 1.6 × 10^18^ cm^−3^. Apparently, GO shows a positive shift of the flat-band potential as compared to the WO_3_ electrode and rather indicates a lower concentration as compared to the N donor.

The band potential alignment of the as prepared GO.WO_3_ vs. NHE shown in Figure 7G illustrates higher activity since its CB edge potential is more active than that of WO_3_ indicating that photogenerated electrons from GO.WO_3_ are easily transferrable than that of WO_3_ via the former formed interface. Alike, the VB edge potential of GO.WO_3_ is less positive than WO_3_, specifying that the lower potential of the former facilitates its holes energetics. Accordingly, sticking a large number of electrons in the conduction band of GO.WO_3_ as well as holes in its valence band increases the life time of e^−^-h^+^ and slow down their recombination to markedly enhance the photocatalytic performance of GO.WO_3_. The electrons may transport into the carbons of GO rather than recombined with the holes in the prevented band. The rest of the electrons on the WO_3_ nanorods surface can react to form reactive oxygen moieties (·${\text{O}}_{2}^{-}$); of reduced lifetime, which react accordingly with 2H^+^ to form •OH. The latter reactive species can also be formed from the reaction of holes with the OH^−^/H_2_O species, to degrade the MB dye more efficiently (Zhang and Yi-Jun, 2016). The two-electron reduction of O_2_ to form H_2_O_2_; as a common role of RGO-constructed composite photocatalysts (Weng et al., 2014), is augmented on the 1%GO.WO_3_ surface. Here, the presence of H_2_O_2_ is determined via using the Ghormley triiodide method (Björkbacka et al., 2015), by which an electron acceptor is donated to form ·OH species. Based on the difference in work function of the GO in favors of that of WO_3_ an electron transfer into the latter from the former is expected, and thus increases the electron density. Rendering the n-type conductivity to WO_3_ surface in front of the p-type of the residual oxygen in GO facilitates the n–p semiconducting boundary interface that causes an enhanced photocatalysis. Accordingly, the electron transfer is found to be efficient in 1%GO.WO_3_ rather than in GO free WO_3_ catalyst. The latter showed high recombination rates of charge carriers of revealed insignificant lifetime, leading to a lower photocatalytic performance. Apparently, our catalyst shows higher photocatalytic efficiency compared with some WO_3_-based graphene composites toward the degradation of the MB dye (Fan et al., 2012; Azimirad and Safa, 2015; Sun et al., 2015; Dinari et al., 2016; Liu et al., 2017). As revealed, their photocatalytic efficiencies are found to be in the range 65–95% within 56–180 min as well as with lower rates than ours.

### Gas Sensing Behavior

**Figure 8:**
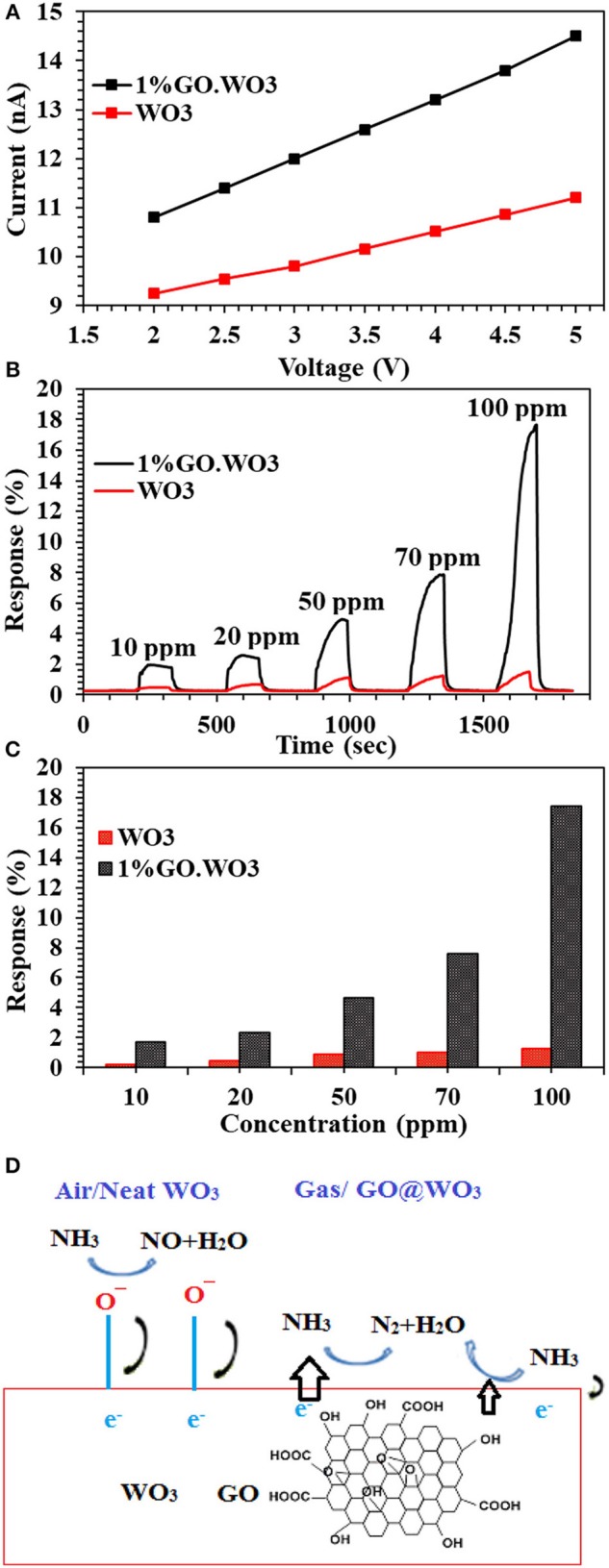
I-V characteristic curves of WO_3_ and 1%GO.WO_3_ catalysts **(A)**, and their response transient to different ammonia concentration (10–100 ppm) at 200^o^C **(B)** response Vs. NH_3_ concentration for WO_3_ and 1 % GO.WO_3_ **(C)** beside the mechanism of NH_3_ gas sensing on the 1%GO.WO_3_ catalyst **(D)**.

The I-V characteristic curves of bare WO_3_ NRs and 1%GO.WO_3_ catalysts shown in Figure 8A indicate an Ohmic contact to ensure the sensing performance of the composite itself and not due to the contact between the composite and the electrode (Morsy et al., 2018). It is obvious that the electrical conductivity of the 1%GO.WO_3_ catalyst is much higher than that of bare WO_3_ NRs. Generally, metal oxides exhibit very poor electrical conductivity, and many strategies are devoted to overcome such poor conductivity. In order to investigate the gas-sensing effect of WO_3_ NRs, and 1%GO.WO_3_, these materials are subjected to different ammonia concentrations (10-100 ppm) at 200°C and the relationship between the sensor's response/recovery and ammonia gas is summarized in Figures 8B,C. The WO_3_ NRs sensor shows no response at room temperature, while 1%GO.WO_3_ shows a moderate sensitivity at room temperature (not shown). For comparison, the response/recovery of WO_3_ NRs and 1%GO.WO_3_ sensors toward ammonia gas evaluated at 200°C is performed as similarly depicted in some literatures (Bittencourt et al., 2006; Guo et al., 2012; Zeng et al., 2012; Chen et al., 2013; Gui et al., 2015; Zhang et al., 2017). The sensitivity of 1%GO.WO_3_ to ammonia gas is estimated to be 2, 2.6, 5.1, 7.9, and 17.6 at 10, 20, 50, 70, and 100 ppm, respectively. For GO free WO_3_, the sensitivity was 0.2, 0.4, 0.89, 1.02, and 1.27 at 10, 20, 50, 70, and 100 ppm, respectively. Therefore, the sensitivity to ammonia processed preferentially on 1%GO.WO_3_ is ~14 times higher than that on WO_3_ NRs sensor. This could be due to the conductivity enhancement as a result of the strong interaction exhibited between partially reduced GO and WO_3_ NRs, which is in accordant with the analysis data of TEM, XPS and Raman. It is also observed that the response increase with increasing ammonia concentration. The sensors response curves show that the resistance of sensors recovers its initial value after ammonia gas interruption, indicating a good reversibility. As a general trend for both sensors, the sensors interact directly with ammonia gas, which may lead to the reduction in the electrical resistivity. Rendering the n–p conductivity enhances the sensing response. Apparently, the numbers of oxygen vacancies formed on the WO_3_ surfaces provide many active sites for the gas sensing reactions. In addition, the formed heterojunction is effectively accelerated the electron transfer and helps reducing the activation energy and enthalpy of the adsorbed gas molecules and thus enhances the sensing response; as shown in Figure 8D. The formed hetero-junction also accelerates the sensing response with the target gas that passes through the interface leading to electronic sensitization via modulation of depletion layers at the heterojunction area.

The principal of semiconductor metal oxide gas sensors depends on the material change in electrical resistance due to the interaction between the target gas and the material. When the sensor subjected to air, the oxygen molecules adsorbed on the conduction band of WO_3_ get ionized to oxygen ions such as ${\text{O}}_{2}^{-}$, O^−^, and O^2−^ through capturing free electrons from the surface of the WO_3_ and result in an electron depletion layer at the WO_3_ surface.

The chemical reaction can be composed as follows;

(6) $$\begin{array}{l} {\text{O}}_{2\left(\text{ads}\right)}+e^{-}\Rightarrow {\text{O}}_{2}^{-} \end{array}$$

(7) $$\begin{array}{l} {\text{O}}_{2\left(\text{ads}\right)}+2{\text{e}}^{-}\Rightarrow {\text{O}}^{-} \end{array}$$

(8) $$\begin{array}{l} {\text{O}}_{2\left(\text{ads}\right)}+4{\text{e}}^{-}\Rightarrow {\text{O}}^{2-} \end{array}$$

These forms of the absorbed oxygen species are function in the operating temperature. The first form, as evidenced by XPS and represents major species, (${\text{O}}_{2,}^{-}$ Equation 1) onsets at room temperature and rests at ~100°C. The second form (O^−^, Equation 2) is acquired in the temperature range of 100–225°C. The third form of adsorbed oxygen species (O^2−^, Equation 3) is excited between 225 and 400°C (Chen et al., 2018). This may explain why the optimum working condition for WO_3_ is approximately around 200°C. During ammonia (reducing gas) exposure, the pre-adsorbed oxygen ions release electrons to the WO_3_ surface, causing a decrease in the depletion layer, thereby decreases the electrical resistance (Urasinska-Wojcik et al., 2016).

(9) $$\begin{array}{l} 2\text{N}{\text{H}}_{3}+3{\text{O}}^{-}\left(\text{ads}\right)\to {\text{N}}_{2}+3{\text{H}}_{2}\text{O}+3{\text{e}}^{-} \end{array}$$

When 1%GO.WO_3_ sensor is exposed to NH_3_ atmosphere, the electron transfer occurs through the formed 1%GO.WO_3_ interface (Figure 8D), whereby both of which can provide effective adsorption sites. Rendering the n-type conductivity to WO_3_ surface in front of the p-type of the residual oxygen in GO facilitates the n-p sensing for enhancing the response. GO acts as a catalytic promoter favoring surface reactions between ammonia gas and the adsorbed oxygen species on the sensor surface. Our results exhibited higher response toward ammonia gas sensing (see Table 1) in a relatively shorter time when compared with some various structural WO_3_ catalysts, GO.WO_3_ composites and when WO_3_ forms heterojunction with other oxides (Wang et al., 2006; Hieu et al., 2011, 2012; Toan et al., 2017; Behera and Chandra, 2018; Prabhu et al., 2018).

**Table 1:** Comparison of ammonia gas sensing on some WO_3_ based catalysts.

**Catalyst name**	**Analyte gas**	**Temperature(^**°**^C)**	**Conc. ppm**	**Response sensitivity**	**Response/recovery time(s)**	**References**
WO_3_ nanorod	NH_3_	225	10	16	145/155 s	Prabhu et al., 2018
SnO_2_-WO_3_ nanofilm	NH_3_	300	50–1,000	1.9–20.3	12/58 s	Behera and Chandra, 2018
WO_3_ nanwire	NH_3_	200, 250, 300	100–1,500	2.39–9.67	16, 7, 15/16, 8, 13 s	Toan et al., 2017
Hemispherical WO_3_/G	NH_3_	R. T.	100	10	–	Hieu et al., 2011
WO_3_ nanofiber	NH_3_	350	50–500	low	20 s	Gui et al., 2015
1%GO.WO_3_ nanorods	NH_3_	200	10–100	17.6	10–15 s	This work

## Conclusion

The highly dispersed 1% GO in WO_3_ hexagonal array heterojunction is successfully synthesized using a template free deposition-hydrothermal route for photocatalytic remediation of the MB (20 ppm) dye and for NH_3_ gas sensing (10–100 ppm). The characterization results conducted using various physicochemical techniques show that the high dispersity of GO onto WO_3_ nanorods surface causes an increase in the observed visible light absorptivity for the 1%GO.WO_3_ composite and consequently decreases both energy band gap and PL intensity. The latter two factors are chiefly responsible for enhancing the MB degradation under visible illumination (in 180 min; 0.0154 min^−1^) via freeing electrons of expanded lifetime, as well as played a role in promotion and enhancing the ammonia gas sensing sensitivity to 17.6%.

## Data Availability Statement

The raw data supporting the conclusions of this manuscript will be made available by the authors, without undue reservation, to any qualified researcher.

## Author Contributions

All authors listed have made a substantial, direct and intellectual contribution to the work, and approved it for publication.

### Conflict of Interest

The authors declare that the research was conducted in the absence of any commercial or financial relationships that could be construed as a potential conflict of interest.
